# Anthropometry-based estimation of body heat capacity in individuals aged 7–69 years: the Size Korea Survey 2010

**DOI:** 10.1038/s41598-018-20872-6

**Published:** 2018-02-06

**Authors:** Duong Duc Pham, Jeong Hoon Lee, Ka Yul Kim, Ji Yeon Song, Ji Eun Kim, Chae Hun Leem

**Affiliations:** Department of Physiology, University of Ulsan College of Medicine, 88 Olympic-Ro 43-gil Songpa-gu, Seoul, Republic of Korea

## Abstract

Although our previously developed anthropometry-based calculation of heat capacity (HC) for adults appeared to be precise and valid, its use in children and adolescents may be associated with bias. This study investigated a large dataset from the Size Korea survey, a national anthropometric survey conducted in 2010, to revalidate our previous HC equation and to develop another one that is appropriate for children and adolescents. We enrolled 12,766 participants aged 7–69 years with body composition data measured by multi-frequency bioelectrical impedance analysis. Age was associated with HC in children aged 7–19 years (R^2^ = 0.58) but not in adults (R^2^ = 0.007). Linear regression was appropriate to describe the relationship between HC and body surface area (BSA) in adults, whereas the regression in children and adolescent was quadratic. The previously developed HC equation had high reliability (intra-class correlation coefficient = 0.995) and predictive power (accurate prediction rate = 86.1%) in the >20 age group. The model composed of sex, body weight, BSA, and BSA^2^ was appropriate for the prediction of HC in young individuals aged 7–19 years. In conclusion, anthropometric-based modelling is a simple, reliable, and useful method for the calculation of HC.

## Introduction

In recent years, human thermoregulation has emerged as a research topic due to its associations with dramatic elevations of heat-related morbidity and mortality worldwide^1^. Some populations such as children, elderly, and obese individuals with metabolic-related diseases are more susceptible to heat stress than others^2–4^. Individual variations in various physiological traits such as sex^5,6^, physical training level^7^, central and peripheral neural regulation^8^, subcutaneous circulation status^9^, and sweat gland number and function^6,9,10^ have been extensively investigated. However, little attention has been paid to body heat capacity (HC), a crucial factor in thermoregulation and heat balancing processes in the human body. Energy expenditure (EE) refers to the energy used by an organism to perform homeostasis and other functions, with heat serving as the main by-product. The accumulation of heat produced via EE that is obtained from external sources such as solar radiation or high ambient temperatures gradually increases the body core temperature (T_core_) and initiates heat regulatory process via heat dissipation to the environment^2^. Each body component has its own specific HC (S_p_HC), the energy required to increase the temperature of 1 kg of material by 1 °C, such as 1, 0.507, 0.299, and 0.201 kcal∙kg^−1^∙°C^−^ for body water, fat, protein, and mineral mass, respectively^11^. Thus, at a given heat load, the magnitude and rate of the increase in T_core_ are not identical between people, and this partly results from individual variations in total body HC.

Unfortunately, the actual measurement of total body HC is complicated and not practical for large-scale studies, and very few equations of HC have been developed^12–15^. The most widely used body weight (BW) estimation method employs a permanent coefficient of 0.83 kcal∙kg^−1^∙°C^−1^ multiplied by BW, resulting in overestimated HC values^14^. In our previous study, the calculated HC value using this S_p_HC, regardless of variations in the proportions of body components, was on average overestimated by 1.66–6.28 kcal∙kg^−1^∙°C^−1^ in comparison to body composition-based estimations^16^. We also suggested a simple calculation for HC that uses and body surface area (BSA), which is useful for estimating HC without body-composition measurement. Although this equation appeared to be relatively accurate because it was developed and validated based on individuals aged ≥20 years, the equation should not be used in younger individuals^16^. This is because our previously developed equation is based on BSA values calculated using the DuBois and DuBois formula, which underestimates BSA in children^17^. However, the DuBois formula was based on a relatively small sample size, whereas other estimations of BSA, such as the Haycock and Mosteller formulae, are more accurate^17,18^. Therefore, whether HC calculated by BSA, determined using the Haycock or Mosteller formulae, is more fitted than that calculated using the DuBois formula should be investigated. Furthermore, extensive validation of the calculation of HC should be performed in a large-scale study and the estimation of HC in younger individuals may become possible.

Size Korea is a nationwide project surveys a large scale of anthropometric indices every 5 or 6 years. The study has been conducted by the Korea Agency for Technology and Standard (KAST) since 1979. The output is a national database for used in the garment and textile industry, ergonomic design, ethnic research, and anthropometric studies^19^. Several ISO standards were employed since 2003 to enhance the accuracy and consistency of the surveys^19^. The most recent Size Korea survey (6^th^) was conducted in 2010 with 14,016 participants aged 7–69 years. The data and results of this study have been accessible to the public since 2013^20,21^. In this 6^th^ collection, body composition analysis (BCA) data were obtained using multi-frequency bioelectrical impedance analysis (MF-BIA). Numerous studies show that MF-BIA is a valid method with high validity and reliability for BCA in epidemiology studies in children^22,23^ and adults^24,25^. The 6^th^ Size Korea survey followed international standard requirements for collecting anthropometric data (ISO 15535) to ensure validity and reliability^19,26^. With the above in mind, this study aimed to analyse data from the Size Korea database to validate our previous predictive equation for HC^16^ in a large population and in younger individuals, and to develop an appropriate calculation of HC for children and adolescents.

## Methods

### Participants

This study was performed on the anthropometric data of 14,016 participants aged 7–69 years downloaded from the official website of the Size Korea project^21^. Those who had missing data for age, sex, BW, height, or body composition were excluded (n = 1,122). We also excluded those with total body mass measured by MF-BIA (the summation of water, fat, protein, and mineral mass) 1% higher or lower than the actual BW measured by a digital scale (n = 128). Finally, a total of 12,766 participants were entered into the analysis. Two-thirds of this pool (n = 8,500; 4,369 males and 4,131 females) were randomly selected for inclusion in the TRAIN group, which is used for predictive equation modelling, whereas the remaining 4,266 participants (2200 males and 2066 females) were entered into the TEST group for validation analysis. For age-specific analysis, the TRAIN and TEST groups were then split into the TRAIN_U20 (aged 7–19 years; 2,609 males and 2,560 females), TRAIN_A20 (aged 20–69 years; 1,760 males and 1,571 females), TEST_U20 (aged 7–19 years; 1,338 males and 1,298 females), and TEST_A20 (aged 20–69 years; 862 males and 768 females) subgroups. The study protocol was approved by the institutional review board of the KAST^26^ and are available to the public and have been used in several publications^20,27^.

### Measurements

Data from Size Korea 2010 were collected while strictly following a standard operating procedure (SOP)^19,26^ in which body water, fat, protein, and mineral mass expressed in kg were measured by multi-frequency BIA using the Inbody 230 and InBody 720 body composition analyser (InBody, Seoul, Korea). The InBody 720 is an eight-polar tactile-electrode MF-BIA device that employs six electronic frequencies (1, 5, 50, 250, 500, and 1000 kHz), whereas the InBody 230 is a portable MF-BIA device with two electronic frequencies (20 and 100 kHz). These MF-BIA devices measure the electrical impedance of five body segments including the trunk, arms, and legs and estimate intracellular water (ICW) and extracellular water (ECW) via low and high-frequency electrical flows, respectively. Total body water (TBW) is calculated by summing ICW and ECW, whereas body fat mass (BFM) is determined by subtracting TBW, estimated protein, and mineral mass from BW. Body fat percentage (PBF) was calculated as body fat divided by BW and multiplied by 100. It has been demonstrated that both the InBody 230 and InBody 720 devices are valid and interchangeable for body composition analysis^28,29^. The measurement protocol was implemented by trained measurers strictly following the guidelines of the manufacturers and the structure of the SOP^19^.

BSA was calculated using standing height and BW based on the DuBois and DuBois^30^, Mosteller^18^, and Haycock^17^ formulae.

DuBois and DuBois EF formula:

1 $${\rm{BSA}}\,({{\rm{m}}}^{2})=0.007184\times {\rm{BW}}\,{({\rm{kg}})}^{0.425}\times {\rm{Height}}\,{({\rm{cm}})}^{0.725}$$

Mosteller formula:

2 $${\rm{BSA}}\,\,({{\rm{m}}}^{2})=\sqrt{\frac{{\rm{Weight}}\,({\rm{kg}})\times {\rm{Height}}\,({\rm{cm}})}{3600}}$$

Haycock formula:

3 $${\rm{BSA}}\,({{\rm{m}}}^{2})=0.0024265\times {\rm{BW}}\,{({\rm{kg}})}^{0.5378}\times {\rm{Height}}\,{({\rm{cm}})}^{0.3964}$$

Body HC calculated using the four-body-compartment model as described previously^11,16^ was used as the reference (HC_Ref):

4 $${\rm{HC}}\,({\rm{kcal}}\cdot ^\circ {{\rm{C}}}^{-{\rm{1}}})=[\begin{array}{c}({\rm{Fat}}\,{\rm{mass}})\times 0.507+({\rm{Protein}})\times 0.299\\ +\,({\rm{Water}})\times 1+({\rm{Mineral}})\times 0.201\end{array}](\mathrm{HC}\_\mathrm{Ref})$$

### Statistical analysis

Pearson’s correlation coefficients were calculated to examine the distribution of HC, BW, BSA, and BSA per HC according to age and the correlation between HC, BW, and BSA in the young (7–19 years) and adult (20–69 years) groups. The independent t-test was used to examine the differences in the characteristics of the TRAIN and TEST groups. Multi-variate regression analysis was performed to develop new predictive equations for HC using the TRAIN, TRAIN_U20, and TRAIN_A20 datasets with sex, BW, BSA, and BSA^2^ as covariates. These newly developed equations and previously developed equation^16^ were then validated against the HC_Ref data, using appropriate test sets including TEST, TEST_U20, and TEST_A20. In this validation analysis, the intra-class correlation coefficient (ICC) was calculated to examine the reliability of predictive equation for HC versus HC_Ref (with a value 0–1), and values ≥ 0.8 denoted acceptable reliability^31,32^. Root mean square error (RMSE) was calculated to examine how HC estimated by predictive equations was close to HC_Ref. A Bland-Altman plot was employed to examine the limits of agreement between HC estimated by predictive equations and HC_Ref. Because a prediction with a 2.5% deviation from the actual value was considered as an acceptable error for body composition measurement^33^ and HC_Ref was calculated based on body composition information, we classified predictive values of HC between 97.5% and 102.5%, <97.5%, and >102.5% of the actual HC_Ref as accurate, under, and over predictions, respectively. Statistical significance was set to a two-tailed p-value <0.05. Data were analysed using R version 3.2.3.

### Data availability

The datasets analysed in this study are available in the Size Korea repository (http://sizekorea.kats.go.kr/02_data/outline.asp).

## Result

Table 1 shows the demographic characteristics, body composition, and HC_Ref values of the TRAIN and TEST groups for the entire age range, the 7–19 year group, and the 20–69 year group. No difference in these parameters between the TRAIN and TEST groups in all age groups and in men and women were observed (Table 1). All three formulae for BSA correlated strongly with each other (r = 0.998, 0.997, and 0.999 for DuBois versus Mosteller, DuBois versus Haycock, and Mosteller versus Haycock formulae, respectively) (Figure S1). However, BSA calculated by the Mosteller and Haycock formulae was slightly better in predicting HC than that estimated using the DuBois formula (simple linear regression: R^2^ = 0.984 and 0.986 versus R^2^ = 0.975 in TRAIN_U20, R^2^ = 0.982 and 0.987 versus R^2^ = 0.960 in TRAIN_A20) (Figure S2). Furthermore, because the Mosteller formula is simple, we employed the Mosteller formula for further analysis. BSA hereafter refers to that estimated by Mosteller formula.

**Table 1:** Demographic characteristic, body composition, and heat capacity by sex in training and test sets.

	Training set		Test set		*p* ^#^
	Men	Women	Men	Women	
*Entire cohort* (n)	4,369	4,131	2,200	2,066	
Age (years)	23.3 (15.0)	23.6 (16.3)	22.7 (14.4)	23.9 (16.9)	0.55
Weight (kg)	58.9 (18.1)	48.6 (12.7)	59.0 (17.8)	48.2 (12.5)	0.66
Height (cm)	161.5 (16.2)	152.2 (12.3)	161.8 (15.7)	151.8 (12.5)	0.92
BMI (kg∙m^2^)	22.0 (4.1)	20.6 (3.6)	22.0 (4.1)	20.6 (3.6)	0.69
BSA (m^2^)	1.6 (0.3)	1.4 (0.2)	1.6 (0.3)	1.4 (0.2)	0.76
Total body water (kg)	33.8 (10.0)	25.4 (5.6)	33.8 (9.8)	25.1 (5.6)	0.61
Body fat mass (kg)	12.9 (6.8)	14.1 (6.1)	13.0 (7.0)	14.0 (6.0)	0.89
Protein mass (kg)	9.1 (2.7)	6.8 (1.5)	9.1 (2.7)	6.7 (1.5)	0.59
Mineral mass (kg)	3.1 (0.9)	2.4 (0.5)	3.1 (0.9)	2.4 (0.5)	0.69
HC_Ref (kcal∙°C^−1^)	43.7 (13.1)	35.0 (8.6)	43.8 (12.8)	34.7 (8.5)	0.64
*Age 7–19 years* (n)	2,609	2,560	1,338	1,298	
Age (years)	13.3 (3.5)	13.2 (3.7)	13.4 (3.5)	13.1 (3.7)	0.84
Weight (kg)	50.5 (17.4)	44.1 (12.9)	51.2 (17.3)	43.5 (12.5)	0.79
Height (cm)	155.2 (17.7)	149.0 (14.0)	156.0 (17.2)	148.6 (14.1)	0.60
BMI (kg∙m^2^)	20.3 (3.9)	19.4 (3.3)	20.4 (4.0)	19.3 (3.1)	0.91
BSA (m^2^)	1.5 (0.3)	1.3 (0.3)	1.5 (0.3)	1.3 (0.2)	0.70
Total body water (kg)	29.2 (9.8)	23.3 (5.7)	29.5 (9.7)	23.0 (5.6)	0.81
Body fat mass (kg)	10.8 (6.6)	12.3 (6.0)	11.1 (6.9)	12.1 (5.8)	0.80
Protein mass (kg)	7.8 (2.7)	6.2 (1.5)	7.9 (2.6)	6.1 (1.5)	0.86
Mineral mass (kg)	2.7 (0.9)	2.3 (0.6)	2.8 (0.9)	2.2 (0.6)	0.81
HC_Ref (kcal∙°C^−1^)	37.5 (12.6)	31.8 (8.8)	38.0 (12.5)	31.5 (8.5)	0.79
*Age 20–69 years* (n)	1,760	1,571	862	768	
Age (years)	38.2 (13.0)	40.7 (14.3)	37.3 (12.8)	42.2 (14.5)	0.65
Weight (kg)	71.4 (10.3)	56.1 (7.7)	71.1 (10.1)	56.2 (7.6)	0.81
Height (cm)	170.8 (6.5)	157.4 (5.9)	170.8 (6.2)	157.2 (6.1)	0.74
BMI (kg∙m^2^)	24.4 (3.1)	22.7 (3.2)	24.4 (3.1)	22.8 (3.2)	0.98
BSA (m^2^)	1.8 (0.1)	1.6 (0.1)	1.8 (0.1)	1.6 (0.1)	0.80
Total body water (kg)	40.7 (4.9)	28.7 (3.2)	40.6 (4.8)	28.7 (3.2)	0.71
Body fat mass (kg)	16.0 (5.9)	17.0 (5.1)	15.9 (6.0)	17.1 (4.9)	0.91
Protein mass (kg)	11.0 (1.4)	7.7 (0.9)	10.9 (1.3)	7.7 (0.9)	0.74
Mineral mass (kg)	3.7 (0.5)	2.7 (0.3)	3.7 (0.5)	2.7 (0.3)	0.81
HC_Ref (kcal∙°C^−1^)	52.9 (7.0)	40.2 (5.0)	52.6 (6.8)	40.2 (5.0)	0.77

Data are presented as mean (SD). BMI, body mass index; BSA^¶^, body surface area calculated by DuBois and DuBois, Mosteller, and Haycock formulae provided almost similar means and SD values (for detail see Supplementary Table S1); HC_Ref, calculated heat capacity based on a four-component model was used as the reference. *p*^#^, *p*-value calculated by independent t-test for comparison between TRAIN and TEST set.

**Figure 1:**
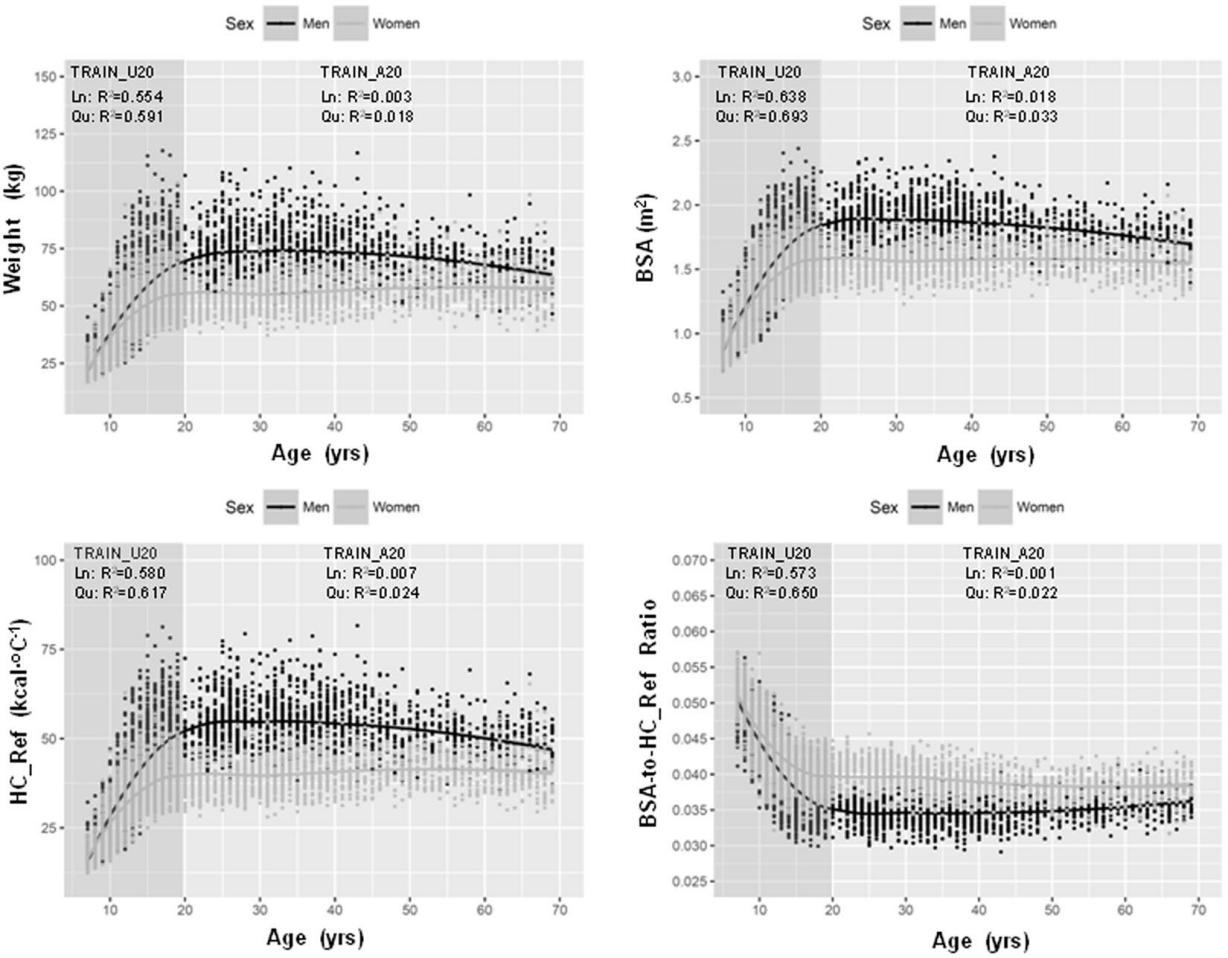
Distribution of body weight, body surface area, heat capacity, and body surface area-to-heat capacity ratio according to age by sex. BMI, body mass index; BSA, body surface area calculated by the Mosteller formula; HC_Ref, calculated heat capacity based on a four-component model according to Pham *et al*.^16^. BSA-to-HC_Ref Ratio was calculated by BSA per HC_Ref.; Ln, simple linear regression model; Qu, simple quadratic regression model; R^2^, R squared or coefficient of determination of the model; TRAIN_U20, training set with age <20 years (darker background); TRAIN_A20, training set with age ≥20 years (brighter background).

**Figure 2:**
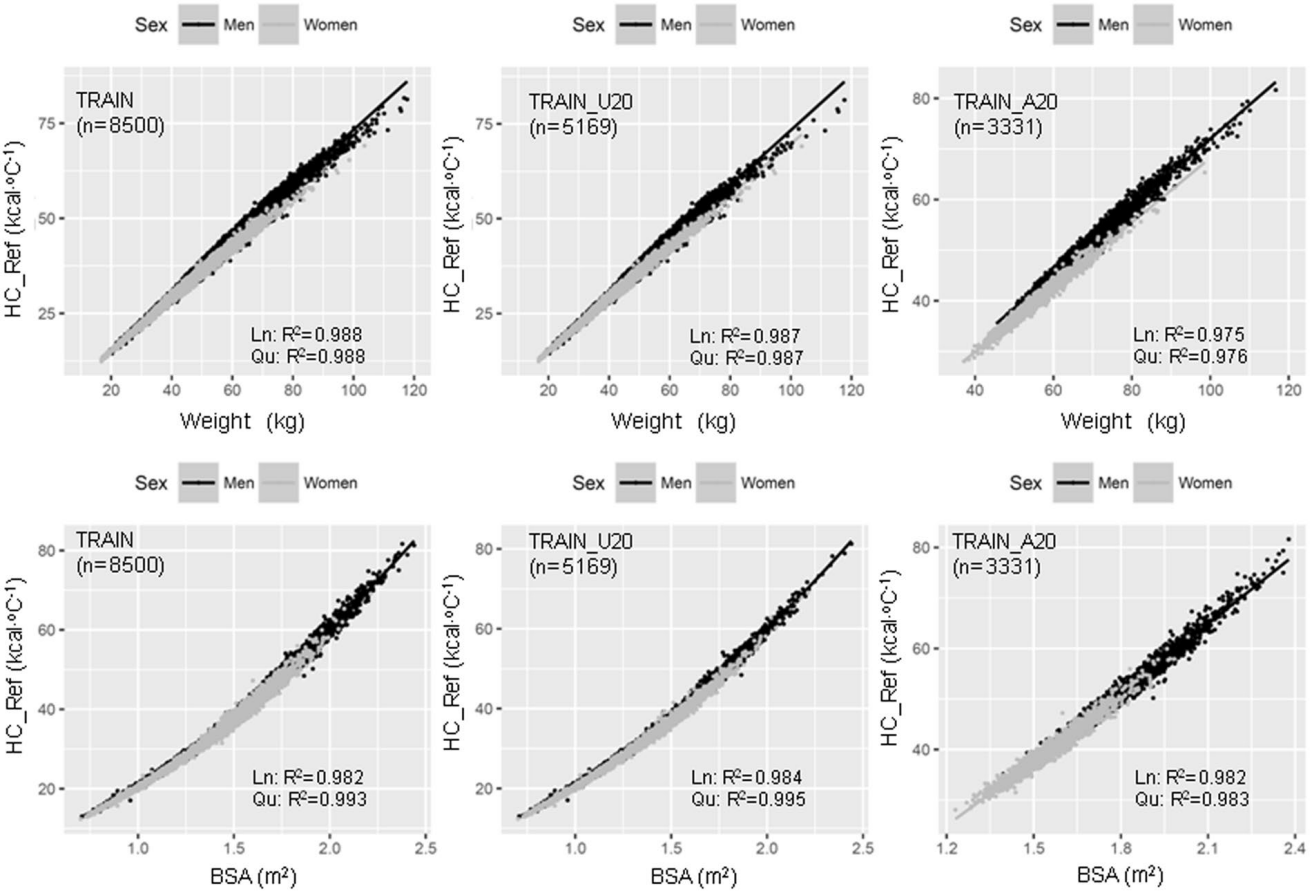
Relationship of body weight and body surface area to heat capacity in the entire group (TRAIN), <20 years group (TRAIN_U20), and >20 years group (TRAIN_A20). BSA, body surface area calculated by the Mosteller formula; HC_Ref, calculated heat capacity based on a four-component model according to Pham *et al*.^5^; Ln, simple linear regression model; Qu, simple quadratic regression model; R^2^, R squared or coefficient of determination of the model.

In the Size Korea data from 2010, two-thirds of participants were aged <20 years. The relationship of age with BW, BSA, HC_Ref and the ratio between BSA and HC_Ref (BSA-to-HC ratio) were different across age groups and sexes. In the <20 years age group, BW, BSA, and HC_Ref dramatically increased whereas the BSA-to-HC ratio reduced with increasing age. These relationships were not linear but quadratic. Using age, linear regression explained 55%, 64%, 58%, and 57%, whereas a quadratic equation explained 59%, 69%, 62%, and 65% of the variation in BW, BSA, HC_Ref, and BSA-to-HC ratio, respectively, in those aged 7–19 years. No substantial correlation between age and BW, BSA, HC_Ref, and BSA-to-HC ratio in adults was detected (Fig. 1). Descriptive analysis showed that there was a sex difference in the relationship between HC_Ref and BW and linear regression was appropriate to demonstrate this relationship in every age group. No sex-specific differences in the correlations between HC_Ref and BSA were found. This relationship was linear in individuals aged ≥20 years and quadratic in the younger population. Using BSA, linear regression explained 98.2%, 98.4%, and 98.2% whereas a quadratic equation explained 99.3%, 99.5%, and 98.3% of the variation of HC_Ref in the entire group, the <20 years group and the >20 years group, respectively (Fig. 2). This trend also appeared with BSA calculated by the DuBois and Haycock formulae (Fig. 2S).

Anthropometry-based predictive equations for HC_Ref were developed based on data from the TRAIN, TRAIN_U20, and TRAIN_A20 datasets using linear (model 1) and quadratic (model 2) regression relationships between BSA and HC_Ref. Model 1 included BW, BSA, and sex, whereas model 2 included BW, BSA, BSA^2^, and sex (Table 2). All models had substantially high coefficients of determination (0.992–0.996). The addition of age and age^2^ into model 1 and model 2 did not improve the coefficient of determination of the models in any age group (data not shown). Based on these results, predictive equations for HC_Ref using model 1 and model 2 for the entire group, the <20 years group, and the >20 years group were developed and encoded as SK_whole_m1, SK_whole_m2, SK_U20_m1, SK_U20_m2, SK_A20_m1, and SK_A20_m2, respectively, in which SK was an abbreviation of Size Korea. The predictive equation for HC in our previous study^16^ was encoded as Leem_Lab_m1 in the present study, in which BSA was calculated using the Dubois formula (Table 3).

**Table 2:** Multivariate regression analysis for predicting HC_Ref according to age groups.

	TRAIN (Aged 7–69 years)	TRAIN_U20 (Aged 7–19 years)	TRAIN_A20 (Aged 20–69 years)
	Regression coefficient (95% CI)	Regression coefficient (95% CI)	Regression coefficient (95% CI)
Model 1	R^2^ = 0.994	R^2^ = 0.994	R^2^ = 0.992
Intercept	−5.915 (−6.147 to −5.683)^‡^	−6.353 (−6.622 to −6.083)^‡^	−8.733 (−9.484 to −7.982)^‡^
Weight	0.439 (0.432 to 0.446)^‡^	0.396 (0.387 to 0.406)^‡^	0.402 (0.388 to 0.415)^‡^
BSA	14.716 (14.316 to 15.116)^‡^	16.298 (15.788 to 16.807)^‡^	17.928 (17.039 to 18.817)^‡^
Sex (Women)	−1.413 (−1.454 to −1.373)^‡^	−1.146 (−1.195 to −1.097)^‡^	−1.635 (−1.72 to −1.549)^‡^
Model 2	R^2^ = 0.996	R^2^ = 0.996	R^2^ = 0.992
Intercept	0.796 (0.489 to 1.102)^‡^	1.458 (1.131 to 1.786)^‡^	1.433 (−0.46 to 3.326)^‡^
Weight	0.277 (0.268 to 0.285)^‡^	0.185 (0.175 to 0.195)^‡^	0.384 (0.371 to 0.397)^‡^
BSA	6.891 (6.454 to 7.328)^‡^	7.269 (6.78 to 7.758)^‡^	6.508 (4.363 to 8.652)^‡^
BSA^2^	5.705 (5.506 to 5.905)^‡^	7.138 (6.905 to 7.37)^‡^	3.556 (2.946 to 4.166)^‡^
Sex (Women)	−1.004 (−1.041 to −0.967)^‡^	−0.859 (−0.897 to −0.82)^‡^	−1.691 (−1.775 to −1.606)^‡^

BSA, body surface area calculated by the Mosteller formula; BSA^2^, body surface area squared; R^2^, the coefficient of determination of the model. Model1 includes body weight, BSA, and sex. Model2 includes body weight, BSA, BSA^2^, and sex. Significant level:^‡^*p* < 0.0001.

**Table 3:** Predictive equations of heat capacity by age groups.

Model	Predictive equation for heat capacity (kcal∙°C^−1^)	Data set
SK_whole_m1	HC = 0.439 × Weight + 14.716 × BSA − 1.413 (if Female) − 5.915	TRAIN
SK_whole_m2	HC = 0.277 × Weight + 6.891 × BSA + 5.705 × BSA^2^ − 1.004(if Female) + 0.796	TRAIN
SK_U20_m1	HC = 0.396 × Weight + 16.298 × BSA − 1.146 (if Female) − 6.353	TRAIN_U20
SK_U20_m2	HC = 0.185 × Weight + 7.269 × BSA + 7.138 × BSA^2^ − 0.859(if Female) + 1.458	TRAIN_U20
SK_A20_m1	HC = 0.402 × Weight + 17.928 × BSA − 1.635 (if Female) − 8.733	TRAIN_A20
SK_A20_m2	HC = 0.384 × Weight + 6.508 × BSA + 3.556 × BSA^2^-1.691(if Female) + 1.433	TRAIN_A20
Leem_Lab_m1	HC = 0.456 × Weight + 14.482 × BSA-1.996 (If Female) − 6.064	Pham *et al*.^16^

HC, heat capacity; SK, models developed based on Size Korea data; Leem_Lab, models developed based on the data of our previous study^16^; TRAIN, whole training set in ages 7–69 years; TRAIN_U20, training set in ages 7–19 years; TRAIN_U20, training set in ages 20–69 years. m1 refers to the model using linear regression of BSA, m2 using quadratic regression of BSA. For SK models, BSA was calculated by the Mosteller formula. For Leem_Lab_m1, BSA was calculated by the DuBois formula.

**Figure 3:**
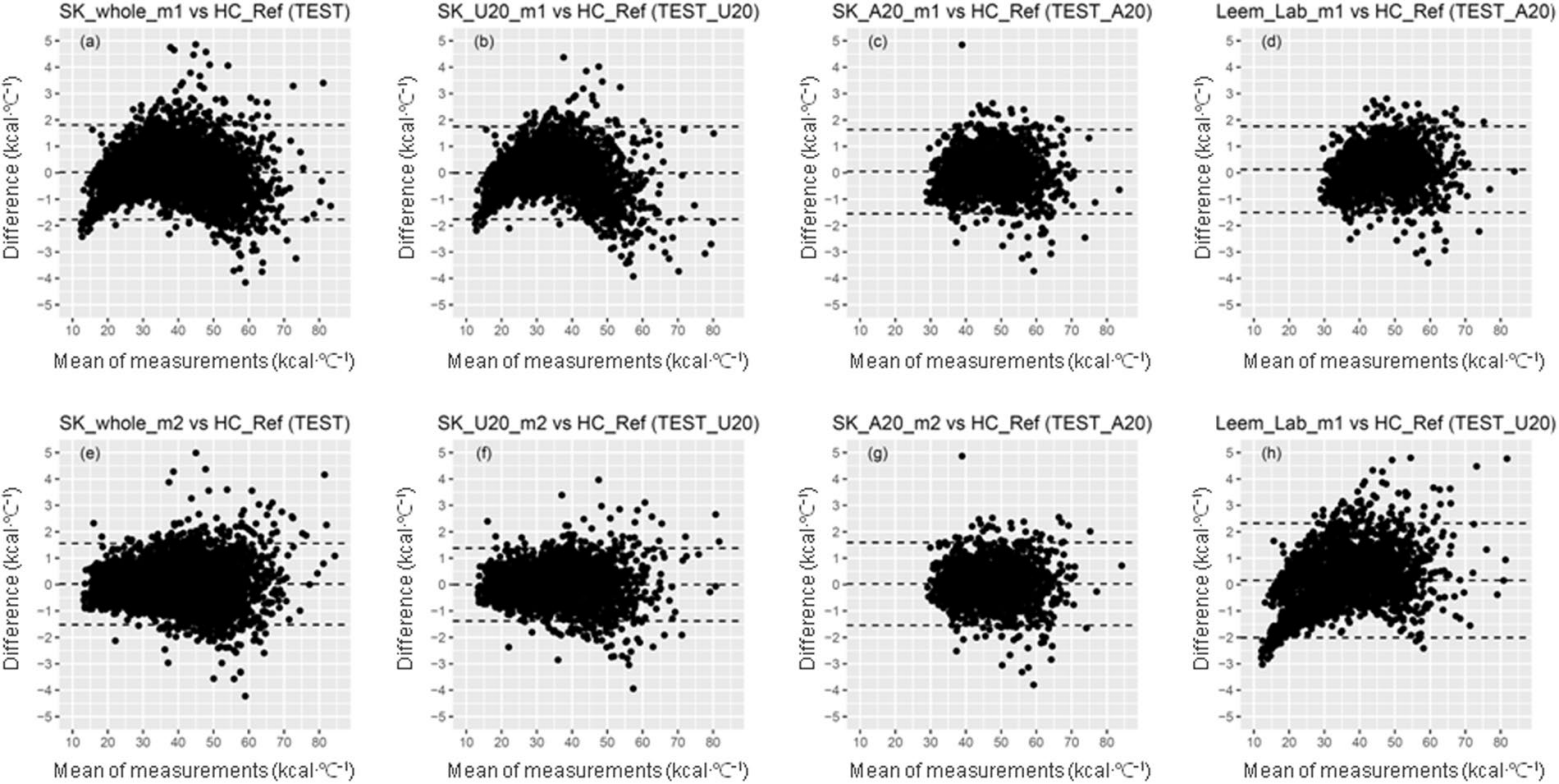
Bland-Altman plots of predictive equations for heat capacity and HC_Ref according to age groups. Validation analysis was performed based on TEST data for (**a**) SK_whole_m1 and (**e**) SK_whole_m2, on TEST_U20 for (**b**) SK_U20_m1, (**f**) SK_U20_m4, and (**h**) Leem_Lab_m1, and on TEST_A20 for (**c**) SK_A20_m1, (**g**) SK_A20_m4, and (**d**) Leem Lab_m1. SK, models developed based on Size Korea data; Leem_Lab, models developed in our previous study^5^; whole, all age groups (7–69 years); U20, age 7–19 years, A20, age 20–69 years; m1 means model using linear regression of BSA, m2 using quadratic regression of BSA. All SK models, BSA calculated by the Mosteller formula. In Leem_Lab_m1, BSA calculated by DuBois formula. For the predictive equation in detail, see Table 3. Data are means of bias and upper and lower limits of agreement.

For equations developed from the Size Korea data, “whole”, “U20”, and “A20” equations were validated using TEST, TEST_U20, and TEST_A20 datasets, respectively, whereas Leem_Lab_m1 was validated using TEST_U20 and TEST_A20 datasets alternately (Table 4 and Fig. 3). Equations based on model 2 were better than those based on model 1 in the whole and <20 years group, with a higher ICC, lower RMSE, and higher percentage of accurate prediction (ICC = 0.998 and 0.998; RMSE = 0.786 and 0.704 kcal∙°C^−1^, accurate prediction = 80.0% and 80.1% for SK_whole_m2 and SK_U20_m2, respectively; ICC = 0.997 and 0.997; RMSE = 0.914 and 0.895 kcal∙°C^−1^, accurate prediction = 73.5% and 68.0% for SK_whole_m1 and SK_U20_m1, respectively) (Table 4). Furthermore, the distribution around the mean measurements were more concentrated in the SK_whole_m2 and SK_U20_m2 models and more bell-shaped in the SK_whole_m1 and SK_U20_m1 models (Fig. 3e,f versus 3a,b).

**Table 4:** Evaluation of HC predictive equations for age groupsEquation.

	Data for validation	r	ICC	RMSE (kcal∙°C^−1^)	Bias (kcal∙°C^−1^)	Lower LOA (kcal∙°C^−1^)	Upper LOA (kcal∙°C^−1^)	Under prediction (%)	Accurate prediction (%)	Over prediction (%)
SK_whole_m1	TEST	0.997	0.997	0.914	−1.775	0.017	1.809	13.0	73.5	13.5
SK_whole_m2	TEST	0.998	0.998	0.786	−1.519	0.021	1.562	9.0	80.0	11.0
SK_U20_m1	TEST_U20	0.997	0.997	0.895	−1.761	−0.006	1.748	16.8	68.0	15.2
SK_U20_m2	TEST_U20	0.998	0.998	0.704	−1.381	−0.002	1.378	9.9	80.1	10.1
SK_A20_m1	TEST_A20	0.996	0.996	0.813	−1.546	0.045	1.636	5.7	87.2	7.1
SK_A20_m2	TEST_A20	0.996	0.996	0.800	−1.544	0.025	1.594	5.0	88.1	6.9
Leem_Lab_m1	TEST_A20	0.995	0.995	0.842	−1.506	0.126	1.758	5.2	86.1	8.7
Leem_Lab_m1	TEST_U20	0.996	0.995	1.116	−2.011	0.156	2.323	19.0	59.3	21.6

SK, models developed based on Size Korea data; Leem Lab, models developed based on the data of our previous study^16^. Test, a dataset including ages 7–69 years; TEST_U20, the test set in ages 7–19 years; TEST_U20, the test set in ages 20–69 years. m1 means a model using linear regression of BSA, m2 using quadratic regression of BSA. BSA was calculated by the Mosteller formula. r, Pearson’s correlation coefficient;

ICC, interclass correlation coefficient = ($\frac{{\rm{Subject}}\,\mathrm{variability}\,}{{\rm{Subject}}\,{\rm{variability}}+{\rm{Measurement}}\,{\rm{error}}}$); RMSE, root mean square error ${\rm{RMSE}}=\sqrt{\frac{\sum {({\rm{Predictive}}{\rm{HC}}-{\rm{HC}}\_{\rm{Ref}})}^{2}}{{\rm{n}}}}$

Bias = mean of difference between predictive HC and HC_Ref; Lower LOA, Lower limit of agreement = mean ‒ 1.96 × SD of difference, Upper LOA, Upper limit of agreement = mean + 1.96 × SD of difference

Accurate prediction, the percentage of participants with predictive HC within 97.5~102.5% of the HC_Ref value.

Underprediction, the percentage of participants with predicted HC lower than 97.5% of the HC_Ref value.

Overprediction, the percentage of participants with predicted HC higher than 102.5% of the HC_Ref value.

For >20 years age group, SK_A20_m1, SK_A20_m2, and Leem_Lab_m1 showed almost the same high reliability and accurate prediction (ICC = 0.996; RMSE = 0.800 vs. 0.842 kcal∙°C^−1^, accurate prediction = 86.1% vs. 88.1%), whereas the Leem_Lab_m1 model had weaker reliability and predictive capacity in the <20 years group (RMSE = 1.116 kcal∙°C^−1^, accurate prediction = 59.3%) (Table 4). A Bland-Altman plot also confirmed this tendency (Fig. 3c,g,d, and h).

## Discussion

This is the first study to describe the relationship of BSA and BW with HC in different age groups. BW was linearly correlated with HC regardless of age, whereas quadratic and linear regressions were appropriate to describe the relationship between BSA and HC among young people and adults, respectively. This study also revealed that anthropometry-based thermoregulation factors including BW, BSA, and HC were age-related in people aged <20 years and that this phenomenon vanished in adults. In the context of temperature regulation, BW, BSA, and HC refer to the heat producing source, heat loss (H_loss_), and heat absorption capacity, respectively.

Children and adolescents may be more susceptible to heat stress than adults because of their greater BSA to body mass ratio. The disadvantages of thermoregulation in children include high relative heat production, low sweating rate, and low cardiovascular insufficiency during exercise^2,34,35^. Therefore, the body of a child tends to dissipate heat via dry heat exchange, rather than evaporation, with the resulting condition that skin temperature (T_s_) is higher than ambient temperature (T_a_). When T_a_ is higher than T_s_, heat absorption from the environment is greater in children than in adults^36^. In the present study, the BSA-to-HC ratio, which may be referred to as H_loss_-to-HC, was high in children, dropped dramatically in adolescents, and varied slightly during adulthood. This phenomenon indicated that modelling of thermoregulation should be performed differently in children and adults. Because our previous predictive equation for HC (Leem_Lab_m1)^16^ was developed based on a population aged ≥20 years, the equation may not be appropriate in children and adolescents. In the present study, Leem_Lab_m1 poorly predicted HC in children and adolescents (TEST_U20) with accurate, under, and over prediction rates of 59.3%, 19.0%, and 21.6%, respectively (Table 4). In line with this, SK_U20_m1, the model employing linear regression between BSA and HC using Size Korea data, also showed a lower reliability and predictive power in the <20 age group. As expected, Leem_Lab_m1 and SK_A20_m1 had high predictive accuracy for adults. This finding suggested that our previously developed predictive equation for HC was highly accurate and that linear regression of BSA to HC should not be applied to children and adolescents.

In the current study, we developed the first predictive equation of HC for children and adolescents, SK_U20_m2, which included sex, BW, BSA, and BSA^2^. This equation was able to explain 99.6% of the variation in HC and had high predictive power (80.1% accurate prediction). For adults, the calculation of HC using BSA (model 1) or BSA^2^ (model 2) provided almost identical results. Thus, SK_A20_m1, SK_A20_m2, and Leem_Lab_m1 are recommended models.

In the current study, we also found that BSA calculated by the Mosteller or Haycock formulae was slightly better at predicting HC. The DuBois formula was developed based on nine individuals with only one child. As such, it tends to underestimate BSA in children^17^. Because the Mosteller formula is the simplest, we selected this calculation of BSA for model development. However, the predictive power and accuracy of predictive equations based on these three BSA formulas were almost identical (data not shown). The calculation of HC for each BSA formula and age group is shown in Supplementary Table S2.

This study has some strengths and limitations. The study was based on a large sample that was collected by a national strictly program following an SOP. As such, its representativeness and external validity are ensured. This is the first attempt to describe the effect of age on HC and its related anthropometric parameters and to develop a predictive calculation of HC for children and adolescents. However, because the Size Korea data include only individuals aged 7–69, this new equation may induce more bias in children below this age range.

In conclusion, the results of this study suggest that age strongly influences the variation in HC and its related parameters in children and adolescents but not in adults. Our previous predictive equation of HC for adult was valid and precise. However, the new equations developed in this study should be used to determine HC based on BW, BSA, and BSA^2^ in children and adolescents.

## Electronic supplementary material

Supplementary Information
