# Supplementary material for: Anthropometry-based estimation of body heat capacity in individuals aged 7–69 years: the Size Korea Survey 2010
Source: Sci Rep. 2018 Feb 6;8:2490. doi: 10.1038/s41598-018-20872-6 (PMC5802818; doi:10.1038/s41598-018-20872-6)
Supplement: Supplementary file 1 — Supplementary Information [file 41598_2018_20872_MOESM1_ESM.pdf]

Supplementary Information

Anthropometry-based estimation of body heat capacity in individuals aged 7–69 years: the Size Korea Survey 2010

Duong Duc Pham[1], Jeong Hoon Lee[1], Ka Yul Kim[1], Ji Yeon Song[1], Ji Eun Kim[1], Chae Hun Leem[1*]

[1]Department of Physiology, University of Ulsan College of Medicine, 88 Olympic-Ro 43-gil Songpa-gu, Seoul, Republic of Korea.

[*] Corresponding author and address correspondence and reprint requests to:

Professor Chae Hun Leem M.D., Ph.D.

Department of Physiology, University of Ulsan College of Medicine/Asan Medical Center, 88 Olympic-Ro 43-gil Songpa-gu, Seoul. The Republic of Korea

Phone: +82-2-3010-4287

Fax: +82-2-3010-8151

E-mail: leemch@amc.seoul.kr, leemch@gmail.com

Author contact information:

Duong Duc Pham: phduongyhct@gmail.com

JeongHoon Lee: biobodhi@gmail.com

Ka Yul Kim: no1gayul@gmail.com

Ji Yeon Song: aster1224@naver.com

JiEun Kim: kje673744@naver.com

Table S1. BSA calculated by DuBois and DuBois, Mosteller, and Haycock formulas

	Training set		Test set		$p^{\#}$
	Men	Women	Men	Women	
Whole group (n)	4369	4131	2200	2066	
BSA_DuBois (m^2)	1.61 (0.32)	1.42 (0.23)	1.62 (0.31)	1.42 (0.23)	0.76
BSA_Mostteler (m^2)	1.61 (0.33)	1.43 (0.24)	1.62 (0.32)	1.42 (0.24)	0.74
BSA_Haycock (m^2)	1.62 (0.33)	1.43 (0.24)	1.62 (0.33)	1.42 (0.24)	0.73
Age <20 yrs (n)	2609	2560	1338	1298	
BSA_DuBois (m^2)	1.47 (0.33)	1.35 (0.25)	1.48 (0.32)	1.34 (0.25)	0.70
BSA_Mostteler (m^2)	1.46 (0.33)	1.34 (0.26)	1.48 (0.33)	1.33 (0.25)	0.72
BSA_Haycock (m^2)	1.46 (0.33)	1.34 (0.26)	1.48 (0.33)	1.33 (0.25)	0.73
Age ≥ 20 yrs (n)	1760	1571	862	768	
BSA_DuBois (m^2)	1.83 (0.14)	1.55 (0.11)	1.82 (0.14)	1.55 (0.11)	0.80
BSA_Mostteler (m^2)	1.84 (0.15)	1.56 (0.12)	1.83 (0.15)	1.56 (0.12)	0.81
BSA_Haycock (m^2)	1.84 (0.16)	1.57 (0.12)	1.84 (0.15)	1.57 (0.12)	0.82

Data are mean (SD). $p^{\#}$, *p* value calculated by independent t-test for comparison between TRAIN and TEST set.

Table 2S. Predictive equations of heat capacity by age groups for each BSA formula

A. BSA calculated by Mosteller formula

Model	Predictive equation for heat capacity ($kcal \cdot {}^oC^{-1}$)	Data set
SK_whole_m1	HC= 0.439×Weight + 14.716×BSA - 1.413 (if Female) - 5.915	TRAIN
SK_whole_m2	HC=0.277×Weight + 6.891×BSA + 5.705× BSA^2- 1.004(if Female)+0.796	TRAIN
SK_U20_m1	HC= 0.396×Weight + 16.298× BSA - 1.146 (if Female) - 6.353	TRAIN_U20
SK_U20_m2	HC= 0.185×Weight+ 7.269× BSA +7.138× BSA^2- 0.859(if Female)+1.458	TRAIN_U20
SK_A20_m1	HC= 0.402× Weight + 17.928× BSA - 1.635 (if Female) - 8.733	TRAIN_A20
SK_A20_m2	HC= 0.384× Weight +6.508× BSA + 3.556× BSA^2-1.691(if Female)+ 1.433	TRAIN_A20

B. BSA calculated by Haycock formula

Model	Predictive equation for heat capacity ($kcal \cdot {}^oC^{-1}$)	Data set
SK_whole_m1	HC= 0.406×Weight + 16.172×BSA - 1.454 (if Female) - 6.383	TRAIN
SK_whole_m2	HC=0.152×Weight + 10.408×BSA + 6.603× BSA^2- 1.009(if Female)-0.084	TRAIN
SK_U20_m1	HC= 0.362×Weight + 17.824× BSA - 1.169 (if Female) - 6.847	TRAIN_U20
SK_U20_m2	HC= 0.035×Weight+ 11.441× BSA +8.234× BSA^2- 0.863(if Female)+0.437	TRAIN_U20
SK_A20_m1	HC= 0.329× Weight + 21.77× BSA - 1.643 (if Female) - 10.802	TRAIN_A20
SK_A20_m2	HC= 0.295× Weight +9.663× BSA + 4.020× BSA^2-1.686(if Female)+ 0.220	TRAIN_A20

C. BSA calculated by DuBois formula

Model	Predictive equation for heat capacity ($kcal \cdot {}^oC^{-1}$)	Data set
SK_whole_m1	HC= 0.496×Weight + 12.002×BSA - 1.348 (if Female) - 4.852	TRAIN
SK_whole_m2	HC=0.422×Weight + 2.898×BSA + 4.545× BSA^2- 0.997(if Female)+1.857	TRAIN
SK_U20_m1	HC= 0.458×Weight + 13.361× BSA - 1.107 (if Female) - 5.203	TRAIN_U20
SK_U20_m2	HC= 0.360×Weight+ 2.538× BSA +5.730× BSA^2- 0.850(if Female)+2.694	TRAIN_U20
SK_A20_m1	HC= 0.494× Weight + 12.789× BSA - 1.639 (if Female) - 5.777	TRAIN_A20
SK_A20_m2	HC= 0.487× Weight +2.304× BSA + 3.116× BSA^2-1.702(if Female)+ 3.406	TRAIN_A20
Leem_Lab_m1	HC= 0.496×Weight + 12.002×BSA - 1.348 (if Female) - 4.852	Pham et al [16]

HC, heat capacity; SK, models developed based on Size Korea data; Leem_Lab, models developed based on the data of our previous study [16]. TRAIN, whole training set with aged 7-69 yrs; TRAIN_U20, training set with aged 7-19 yrs; TRAIN_U20, training set with aged 20-69 years. m1 means model using linear regression of BSA, m2 using quadratic regression of BSA.

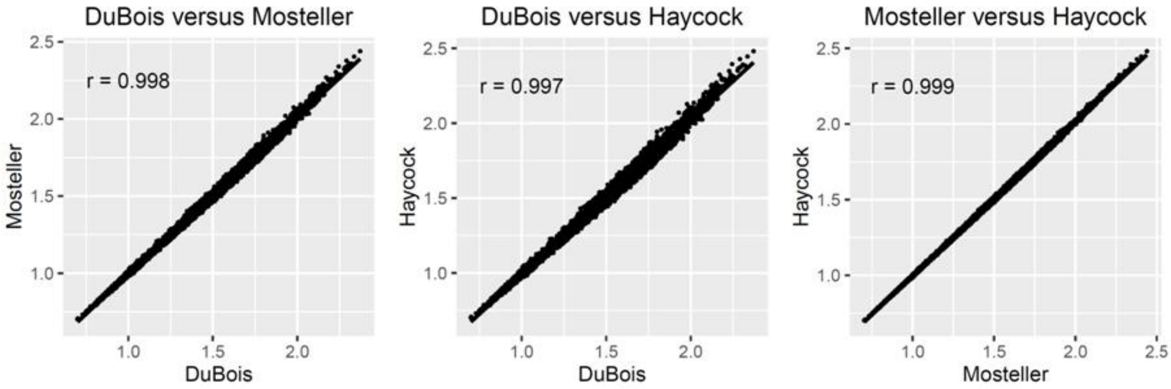

Figure S1. Correlation between three BSA formulas

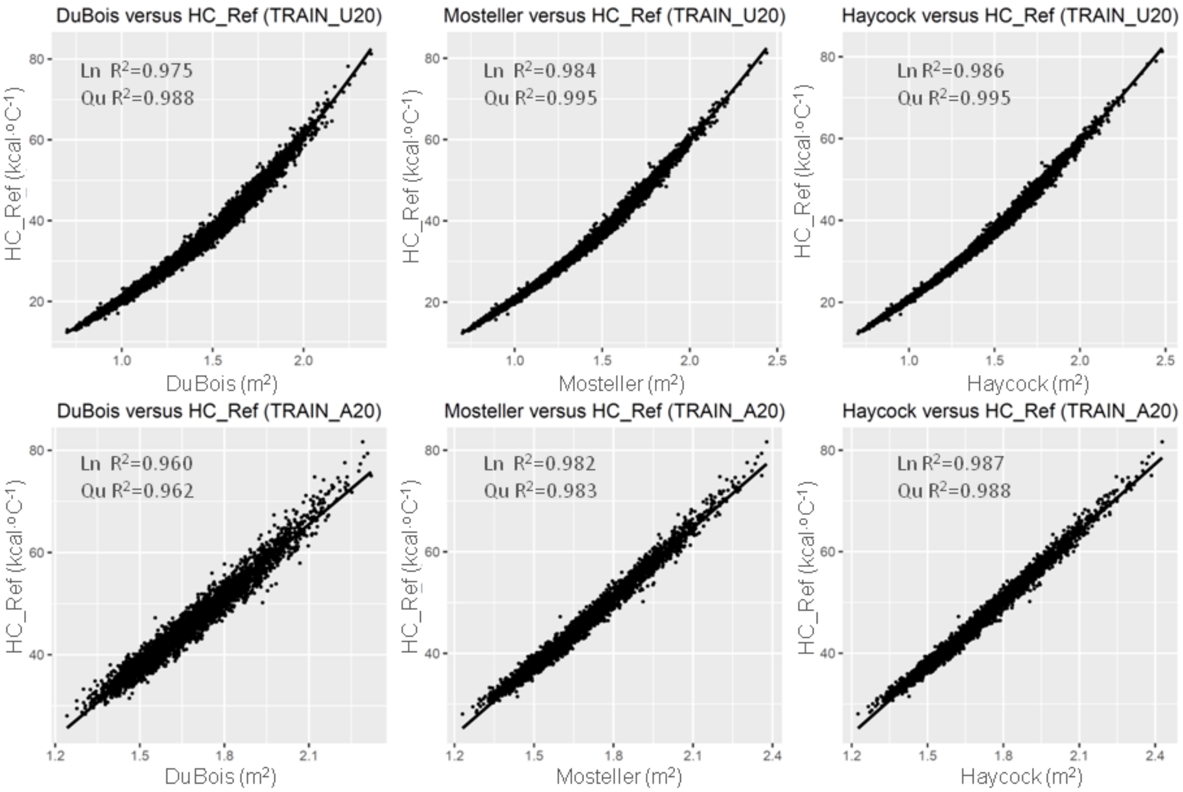

Figure 2S. Relationship of body surface area calculated by three formulas to heat capacity in under 20s (TRAIN_U20), and above 20s (TRAIN_A20). HC_Ref, calculated heat capacity based on a four-component model according to Pham et al [ref]; Ln, simple linear regression model; Qu, simple quadratic regression model; R^2, R squared or coefficient of determination of the model.
